# New Chaotic Reality: Creative Writing Workshops for Long COVID Patients

**DOI:** 10.1007/s10912-024-09891-9

**Published:** 2024-09-09

**Authors:** Ed Garland

**Affiliations:** Department of English and Creative Writing, Aberystwyth University, Aberystwyth, UK

**Keywords:** Long COVID, Creative writing, Chronic illness, Wellbeing

## Abstract

In a widely cited 2017 study, Robinson et al. (2017) found that ‘emotionally expressive’ writing makes physical wounds heal faster when compared to writing that did not engage the emotions. The Writing Long COVID project at Aberystwyth University engaged similar territory in a recent pilot study. Participants’ writing activities explored how literary production can affect a person’s experience of this new chronic condition, as well as contribute to our understanding of its symptoms. In this short essay, I show how we designed a course of short-duration online workshops that increased accessibility for people with Long COVID-related fatigue. I also argue that future Long COVID creative activities should let their timing, venue, content, and structure be influenced by the preferences of the Long COVID patient. The preliminary study suggests that the traditional parameters of the writing workshop, including its duration, could deter participation in potentially beneficial creative activities.

## Introduction

The Writing Long COVID project at Aberystwyth University introduced a new framework for creative writing workshops. Half-hour durations and small group sizes made these workshops more accessible to people who live with fatigue and brain fog. This brevity is less daunting than a more standard one or two hour duration and limits the participants’ exposure to post-exertional malaise. The small group size of between two and six participants ensures everyone who wants to speak is given a chance to do so. Thus, the provision of specially structured workshops through social prescription could increase the likelihood of participation in an activity that promotes wellbeing for chronically ill people.

This article describes how the workshops were delivered and then discusses how their innovative form could shape therapeutic creative writing practises and help to challenge plot-driven, climax-oriented creative writing orthodoxies by deprivileging the sense of resolution in the dynamics of a story. The title ‘New Chaotic Reality’ is a phrase that emerged during the workshops, which both describes how the participants viewed their illness and evokes the kind of experience that Long COVID workshops need to consider.

## Creative writing and long COVID

Writing affects a person’s wellbeing and can influence physical changes in the body. For example, in a 2017 study, Robinson et al. found that ‘emotionally expressive’ writing makes physical wounds heal faster when compared to writing that did not engage the emotions (Robinson et al. 2017). The Writing Long COVID project aimed to use emotionally expressive writing to explore how creative production can affect a person’s experience of this new chronic condition.

Long COVID is usually defined as the presence of ‘prolonged symptoms following infection with SARS-CoV-2 that are not explained by an alternative diagnosis’ (Greenhalgh 2022). In the UK, there are 1.9 million people living with this condition (ONS 2023). The wide range of Long COVID symptoms includes brain fog, fatigue, shortness of breath, loss of smell, aches, insomnia, dizziness, and depression (NHS 2023). Not everyone who has the condition will have all of these symptoms, and there is not yet a reliable treatment. As the charity Long COVID SOS makes clear, Long COVID patients had to take long-term collective action to have the condition recognised by the public and the healthcare authorities as a serious, life-changing condition, whose effects are often made worse by ignorance and suspicion: ‘sufferers may be unable to get support from family and friends who do not understand why they are ill for so long, and many are put under pressure to return to work or otherwise face a loss of sickness benefit’ (Long Covid SOS 2023). Some of the most frequent recommendations for managing the condition include ‘lifestyle changes’ such as giving up smoking and learning about ‘simple strategies’ people might use to ‘improve their mood, sleep, and quality of life’ (Vasu 2022).

## Trialling the workshops

The project took place in summer 2022, as part of Aberystwyth University’s Centre for Creativity and Wellbeing, which researches the relationship between creative writing and public health. One of the centre’s aims is to embed creative writing activities into local public healthcare provisions for managing chronic illness.

In this pilot study, there were four workshops over 2 weeks in summer, online via Teams in the early evenings. In each session, participants were given two sets of writing prompts and imaginative scenarios, with a short time in which to respond to these prompts in writing. Then, they were invited to share what they had written by reading the work aloud and were offered positive feedback, plus discussion amongst themselves, which focussed on highlighting how the writing expressed the particularities of the Long COVID experience. Follow-up discussion via email was invited. The prompts were designed to relate to physical and imaginative aspects of their specific illness and to include both traditional and non-standard creative writing workshop prompts. In week one, participants were asked to describe their Long COVID symptoms and to create a character related to that description. In week two, they were asked to imagine setting off on a journey but not necessarily returning. These first two weeks’ prompts are more or less traditional in a creative writing workshop. Week three’s prompts related to visions of the future, and week four addressed the topic of air, both of which emphasised in a less traditional manner the differences chronic illness makes to a person’s relationship with time and the environment. The element of ‘chaotic reality’ was reinforced by reminding the participants that they did not have to conform to pre-existing, able-bodied expectations about creative writing orthodoxy. Both before and after the four workshops, the participants responded to a short survey about their experience of the writing activity, and their experience of their illness.

## Participants’ responses to the workshops

At the end of one session, participant Louise mentioned that the half-hour timeframe made the workshop less daunting to consider attending and less tiring to attend than a normal duration workshop would have been.^1^ In other words, the half-hour duration made the activity accessible. She expanded on this idea in written feedback after the course finished:

[on one occasion] both myself and the other participant were struggling more than usual with our symptoms and each said if the session had been longer than 30 min we both would have had to send our apologies and been unable to attend. As it was, the short and succinct approach to the session made us feel that we could just about attend and engage. (Post-workshop written feedback 2022)

So the short duration, despite being unorthodox by the conventions of creative writing, felt appropriate to this participant. By saying she could ‘just about’ attend, Louise here highlights how a shorter duration lowers the barrier to access enough to enable her to enjoy the activity. A more traditional one or two hour workshop would have been out of her reach. The online nature of the sessions also enabled access. Participants could attend from wherever they felt most comfortable—couch, chair, or bed.

Another positive result of the workshops was that participants felt a sense of ‘catharsis’—both in the writing itself, and in the process of sharing it with the group (Postworkshop written feedback 2022). One of the frustrations of Long COVID is that it is difficult to communicate such a profound change in daily experience and attempts to do so often encounter a culture of scepticism and disbelief. The participants explored these tensions in imaginative ways. One of them wrote that their life was like living inside a snow globe, while the world outside seemed to continue as normal. As Louise put it in her post-course feedback, the sessions were ‘a great tool to deal with my frustrations’—one of which was the loss of ‘bodily autonomy’ that is one of Long COVID’s key features (Post-workshop written feedback 2022). In a widely-circulated article in August 2020, Ed Yong described how the people who are closest to a Long COVID patient can react negatively to their sudden loss of bodily autonomy:

long COVID almost always comes with an equally debilitating comorbidity of disbelief. Employers have told long-haulers that they couldn’t possibly be sick for that long. Friends and family members accused them of being lazy. Doctors refused to believe they had COVID-19. (Yong 2023)

In 2022, when the workshops took place, people with Long COVID had made progress through collective action in somewhat reducing the prevalence of these attitudes—for example, by forming the awareness-raising charity Long COVID SOS. But the Writing Long COVID participants clearly still encountered such attitudes in their day-to-day lives. Thus, another one of the workshop’s positive effects was that it provided a supportive atmosphere and fostered a kind of solidarity between people who face similar challenges. Humorous writing also enabled this sense of solidarity and catharsis. For example, when asked to describe characters based on their Long COVID symptoms, Louise created an amorphous ‘faceless’ being, and Carrie presented a man called the ‘overprivileged douchebag.’ Humour was always present to some degree in the workshops but especially so when these characters were discussed, and the shared laughter emphasised the fact that creative writing engages the body—particular word choices can cause us to involuntarily or semi-voluntarily exercise our muscles and our respiratory tracts. Perhaps chronic conditions have their own kind of laughter, rooted in the tensions between disbelief and acceptance, impatience, and persistence.

Alongside the affirmation of experiential similarity between participants, the workshop also enabled them to recognise differences in their experiences of Long COVID. Responses to the prompt about air highlighted some of these differences. Participants were asked to respond to the phrase ‘the air today is…’. While Carrie wrote about thin air, and the idea of ‘air hunger’, Louise described ‘thick’ air, related to a sense of nausea. This conceptual disparity illustrates Sarah McLusky’s description of breathlessness as ‘a complex experience that is difficult to articulate, and clinical language can obstruct understanding’ (McLusky 2023). The variety of creative articulations of respiration in the Writing Long COVID workshops helps us to understand the subjectivity of breathlessness—while both participants imagine it as a material problem, located in the air, one of them conceives of it as an excess, while the other views it as a deficiency.

The participants in this study already had an interest in literature and valued writing as an activity in itself. Bearing these preferences in mind, we can now briefly consider how the Long COVID Writing project might influence literary production in the context of health.

## Writing, workshops, and health

A key moment in the history of literature and health occurred in Russia in 1849. Before his exile to Siberia that year, Dostoyevsky wrote two letters to his brother in which he used the term ‘nerves’ to refer to his experiences of epilepsy, and of what we nowadays might call anxiety and stress:

I am afraid of working [i.e. writing] very much. This work […] always used to exhaust me by acting on my nerves […]. I conclude that my nerves are going to pieces. When such a nervous time came upon me formerly, I made use of it for writing – always in such a condition you write better and more, but now I restrain myself in order not to finish myself off altogether. (Rice 1985)

In these sentences, Dostoyevsky identifies something that the Writing Long COVID project had to consider: the fact that chronic illness gives people a great deal to write about, and the fact that the process of writing, especially about difficult experiences, requires significant exertion. There is always a tension between the need to communicate and the physical costs of that communication. In Long COVID specifically, the exertion required for imaginative work like creative writing could have a negative impact on fatigue. Brain fog can make sentences difficult to compose. Large amounts of text on the screen can be difficult to read.

Some subjects are harder to write about than others. Thus, another advantage of the workshops’ half-hour duration is that it limits exposure to creative difficulties.

There was only enough funding for a short course of four workshops, but we were still able to question what kinds of narratives might arise from, and relate to, the experience of chronic illness. As van Hout, van Rooden, and Slatman argue in a recent article about narrating chronic pain, ‘studies have shown that more flexible, intermodal and interactive types of narrating, as afforded by the internet and social media, might facilitate pain storytelling’ (van Hout et al. 2022). The reason given is that ‘chronic pain resists coherent, plotdriven’ narratives because it has no end (van Hout et al. 2022). Likewise, for many people, Long COVID is endless, and thus future workshops could explore narratives that prioritize a sense of ongoingness in various ways, instead of stories that build to a climax and offer a sense of resolution. Future workshops could also increase interactivity and flexibility by introducing a non-synchronous element, such as a forum or social media thread, within which participants can explore the writing prompts and engage with the course materials at a pace that suits them. We might then have a writer-oriented workshop, to complement their patient-oriented care.

## Conclusion

While we await the appearance of a cure for Long COVID, we need to remember that chronic illness fundamentally alters the way people experience the world. The written articulation of these fundamental changes is an ongoing collective project. Initiatives such as PhotoVoice, in which individual members of various communities are given cameras to make art and curate exhibitions based on their life experiences, offer a model for how Long COVID patients could advocate for themselves through artistic practice (I thank one of this essay’s anonymous peer-reviewers for this insight). Long COVID itself is perhaps subject to more scepticism and debate than other similar conditions, and creative writers are well-placed to advocate on behalf of their global community through innovative textual productions that focalise social experience through the complexities of disability. To do this properly, people need to be given the chance to articulate their experiences within a workshop that takes account of their priorities. In other words, a workshop that derives its timing, venue, content, and structure, from the preferences of the Long COVID patient.

## Endnotes

^1^ All participants’ names have been pseudonymised.

## References

[CR1] Greenhalgh, Trisha. 2022. Long COVID: An update for primary care. BMJ 378: e072117. https://www.bmj.com/content/378/bmj-2022-072117. Accessed 16 October 2023. 10.1136/bmj-2022-072117 36137612

[CR2] Long Covid SOS. 2023. Our story. https://www.longcovidsos.org/our-story. Accessed 16 October 2023.

[CR3] McLusky, Sarah. 2023. A pandemic of breathlessness? Life of breath. https://lifeofbreath.webspace.durham.ac.uk/a-pandemic-of-breathlessness/. Accessed 16 October 2023.

[CR4] NHS. 2023. Long-term effects of COVID-19 (Long COVID). https://www.nhs.uk/conditions/covid19/long-term-effects-of-covid-19-long-covid/. Accessed 16 October 2023.

[CR5] Office for National Statistics. 2023. Prevalence of ongoing symptoms following coronavirus (COVID-19) infection in the UK: 30 March 2023. https://www.ons.gov.uk/peoplepopulationandcommunity/healthandsocialcare/conditionsanddiseases/bulletins/prevalenceofongoingsymptomsfollowingcoronaviruscovid19infectionintheuk/30march2023. Accessed 16 October 2023.

[CR6] Rice, James. 1985. *Dostoyevsky and the healing art: An essay in literary and medical history*. Ann Arbor: Ardis.

[CR7] Robinson, Hayley, Paul Jarrett, Kavita Vedhara, Elizabeth Broadbent. 2017. The effects of expressive writing before or after punch biopsy on wound healing. *Brain, Behaviour, and Immunity *61: 217-227. 10.1016/j.bbi.2016.11.025 10.1016/j.bbi.2016.11.025 27890660

[CR8] Van Hout, Femke, Aukje van Rooden, Jenny Slatman. 2022. Chronicling the chronic: Narrating the meaninglessness of chronic pain. *Medical Humanities* 49(1): 1-8. 10.1136/medhum-2021-012331 10.1136/medhum-2021-012331 PMC9985763 35851264

[CR9] Vasu, Thanthullu. 2022. *Managing LONG COVID Syndrome*. Harley: TFM Publishing.

[CR10] Yong, Ed. 2023. Long-Haulers Are Redefining COVID-19. The Atlantic. https://www.theatlantic.com/health/archive/2020/08/long-haulers-covid-19-recognitionsupport-groups-symptoms/615382/. Accessed 16 October 2023.

